# A Single Naturally Occurring 2’-O-Methylation Converts a TLR7- and TLR8-Activating RNA into a TLR8-Specific Ligand

**DOI:** 10.1371/journal.pone.0120498

**Published:** 2015-03-18

**Authors:** Stephanie Jung, Tina von Thülen, Viktoria Laukemper, Stephanie Pigisch, Doris Hangel, Hermann Wagner, Andreas Kaufmann, Stefan Bauer

**Affiliations:** 1 Institut für Immunologie, Philipps-Universität Marburg, BMFZ, Marburg, Germany; 2 Institut für Medizinische Mikrobiologie, Immunologie und Hygiene, Technische Universität München, München, Germany; University Hospital Zurich, SWITZERLAND

## Abstract

TLR7 and TLR8 recognize RNA from pathogens and lead to subsequent immune stimulation. Here we demonstrate that a single naturally occurring 2’-O-methylation within a synthetic 18s rRNA derived RNA sequence prevents IFN-α production, however secretion of proinflammatory cytokines such as IL-6 is not impaired. By analysing TLR-deficient plasmacytoid dendritic cells and performing HEK293 genetic complementation assays we could demonstrate that the single 2’-O-methylation containing RNA still activated TLR8 but not TLR7. Therefore this specific 2’-O-ribose methylation in rRNA converts a TLR7 / TLR8 ligand to an exclusively TLR8-specific ligand. Interestingly, other modifications at this position such as 2’-O-deoxy or 2’-fluoro had no strong modulating effect on TLR7 or TLR8 activation suggesting an important role of 2’-O-methylation for shaping differential TLR7 or TLR8 activation.

## Introduction

Toll-like receptors (TLRs) belong to a set of innate immune receptors that sense microbial associated molecular patterns (MAMPs) and activate immune and non-immune cells [1]. One subfamily of TLRs that consists of TLR3, 7, 8, 9 and 13 is activated by nucleic acid in endosomal / lysosomal compartments [2,3]. TLR3 recognizes double stranded (ds) RNA [4], TLR13 senses bacterial 23s rRNA [3,5] and TLR9 is a receptor for unmethylated CpG-DNA [6]. TLR7 and TLR8 are activated by nucleoside analoga (e.g. imidazoquinolines such as imiquimod or resiquimod), poly-U or GU-rich single stranded (ss) oligoribonucleotides (ORN), viral RNA and siRNA [7–12]. Importantly, activation of TLR7 and TLR8 induces upregulation of costimulatory molecules and cytokine production [10]. In humans, TLR7 is only expressed in plasmacytoid dendritic cells and B cells, whereas TLR8 is found in monocytes, myeloid dendritic cells and regulatory T cells [13,14]. In general, TLR7 activation induces strong type I interferon production in human plasmacytoid dendritic cells, whereas TLR8 stimulation mainly leads to proinflammatory cytokine production (e.g. IL-6 and others) from human monocytes and myeloid dendritic cells [10,15]. In mice, TLR7 is broadly expressed in various cell types such as plasmacytoid dendritic cells, myeloid dendritic cell, B cells and monocytes / macrophages. Murine TLR7 stimulation leads to type I interferon secretion from plasmacytoid dendritic cells whereas proinflammatory cytokines are secreted by myeloid dendritic cell and monocytes / macrophages. Murine TLR8 is non-functional per se, but the addition of poly-T deoxynucleotides (poly-dT) *in vivo* and *in vitro* induces and enhances ligand specific cytokine production [16].

Due to cell type specific receptor expression and differential cytokine production, specific activation of either TLR7 or TLR8 may be important for shaping the cytokine milieu and subsequent immune response in therapeutic intervention. Accordingly, synthetic TLR7- or TLR8-specific imidazoquinolines have been used preclinically, however systemic application is generally not applicable due to strong side effects [17–19]. In contrast, TLR-activating immunostimulatory ORN have less side effects, but for specific TLR7 or TLR8 activation only limited ORN sequences are available [15,20].

We therefore investigated if single RNA modifications would influence ligand specificity for TLR7 or TLR8. We and others have shown that synthetic RNA with multiple 2’-O-methylations antagonize TLR7- and TLR8-mediated cytokine secretion [21–24]. Also other RNA modifications such as conversion of uridine to pseudouridine and methylation of bases can lead to loss of TLR7 and TLR8 mediated immune stimulation [21]. In addition, naturally occurring RNA modifications can modify RNA recognition by TLRs. Accordingly, 2’-O-methylation of tRNA at a conserved guanosine in a dinucleotide motif abolishes TLR7 activation and N6-adenosine-methylation of bacterial 23s rRNA renders it inactive against TLR13 activation [3,25–27]. Here we demonstrate that a single 2’-O-methylation within a synthetic 18s rRNA derived RNA sequence prevents IFN-α production, whereas IL-6 secretion is not impaired. Other modifications at this position such as 2’-O-deoxy or 2’-fluoro (2’-F) had no negative effect on TLR7 or TLR8 activation. Therefore a single 2’-O-methylation converts a TLR7- / TLR8-activating RNA to a TLR8-specific ligand.

## Results and Discussion

### Differential induction of activation markers by non-modified and 2’-O-methylated synthetic RNA derived from 18s rRNA

**Fig 1:**
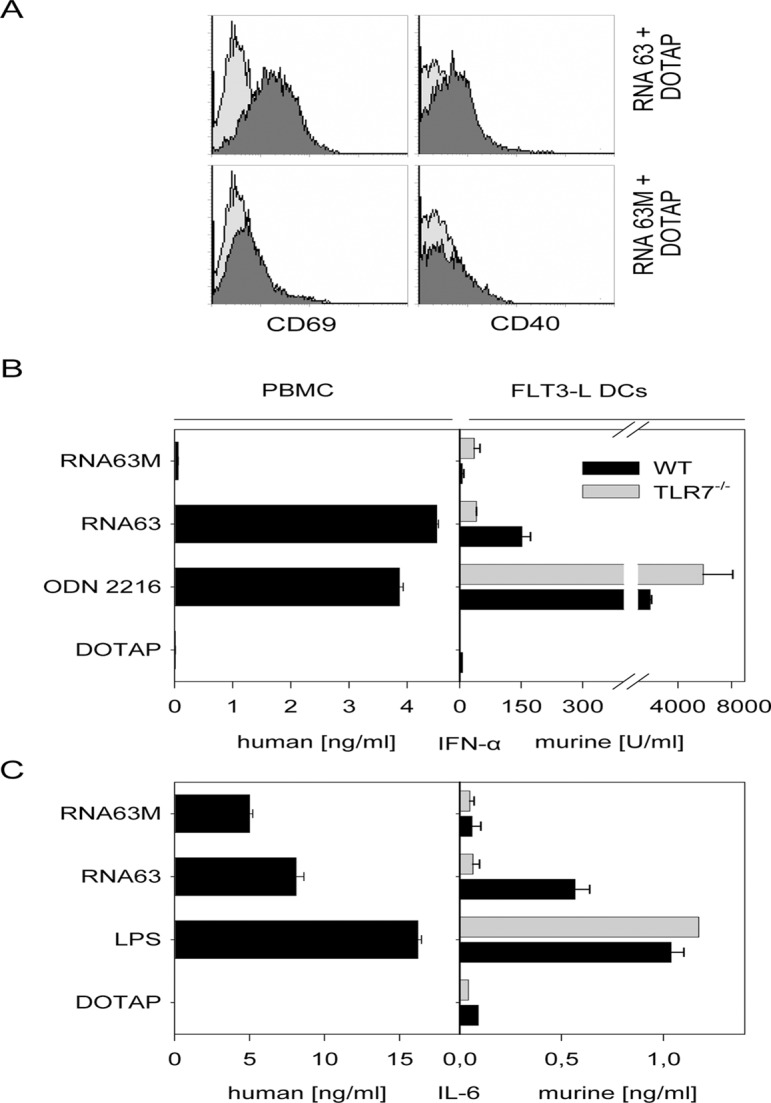
Differential induction of activation markers and cytokines by non-modified and 2’-O-methylated synthetic RNA. (A) Murine FLT3L-DCs were stimulated with RNA63 or RNA63M complexed to DOTAP (dark grey) and stained for CD69 or CD40 as indicated. Unstimulated cells (same histogram data for both RNA stimuli) served as negative control (light grey). (B, C) Human PBMC (B) and murine WT or TLR7-deficient FLT3-L induced DCs (C) were stimulated with RNA63 (2 μg/ml), RNA63M (2 μg/ml) and ODN2216 (1 μg/ml) or LPS (100 ng/ml) and IFN-α or IL-6 measured by ELISA after incubation for 20 hours. For panel A, B and C one representative experiment of at least three independent experiments are shown (n = 2 ±SD).

To investigate the influence of 2’-O-ribose methylation on immunostimulatory activity of rRNA, we synthesized two oligoribonucleotides (ORN) derived from the 18s rRNA differing in their 2’-O-ribose methylation status. The selected ORN RNA63 was derived from nucleotides 1488–1499 of the 18s rRNA (5’ CAGGUCUGUGAU). At position 1490 the natural rRNA contains a 2’-O-methylated guanosine [28,29], which is mimicked by RNA63M (5’ CA***Gm***GUCUGUGAU). For RNA stimulation of murine primary immune cells we generated murine dendritic cells (DC) by cultivation of bone marrow cells with FMS-like tyrosine kinase 3-Ligand (FLT3-L) for 8 days. Under these culture conditions a mixture of plasmacytoid and myeloid dendritic cells grows (termed FLT3-L induced DCs) that induces type I interferon and proinflammatory cytokines upon TLR7 stimulation. FLT3-L induced DCs from WT mice were stimulated with RNA63 and RNA63M complexed to DOTAP that protects RNA from RNases and facilitates RNA uptake [30]. Interestingly, RNA63 induced upregulation of murine activation markers CD69 or CD40 (Fig. 1A), whereas RNA63M did not. This difference in stimulatory potential was not due to differences in ORN uptake since both ORNs colocalized within immune cells (data not shown). We further tested ORN-mediated cytokine production in WT or TLR7-deficient murine FLT3-L induced DCs. Of note, RNA63 stimulated FLT3L-DCs produced IFN-α and IL-6 in a TLR7 dependent manner (Fig. 1B, C). IFN-α induction by RNA63M was absent supporting the view that TLR7-mediated IFN-α production was abrogated by the single 2’-O-methylation of the modified guanosine at position 3. This observation is in line with reports that describe an antagonistic function of 2’-O-methylated RNA on TLR7-mediated activity [24–27,31].

We also investigated the immunostimulatory potential of RNA63 and RNA63M in human immune cells and therefore isolated human peripheral mononuclear cells (PBMCs) from human buffy coats (see Material and Methods section). These preparations contain lymphocytes, monocytes and plasmacytoid / myeloid dendritic cells. In these cell preparations, stimulation of nucleic acid sensing TLRs leads to a strong IFN-α production by plasmacytoid dendritic cells, whereas proinflammatory cytokines are produced by monocytes and myeloid dendritic cells.

The PBMCs responded to RNA63 mediated stimulation with IFN-α and IL-6 production (Fig. 1B, C). Importantly, RNA63M induced no IFN-α in human PBMCs, but in contrast to murine FLT3L-DCs IL-6 was secreted. Since murine TLR8 is not active per se [8,16], the RNA63 driven immunostimulatory activity in murine cells is mediated only by TLR7. Since human TLR7 and TLR8 differ in their target cell selectivity and cytokine induction profile we hypothesized that RNA63 mediated IFN-α production is TLR7 dependent, whereas production of proinflammatory cytokines is mediated by human TLR8.

### A single 2’-O-methylation converts the TLR7/8 ligand RNA63 into a TLR8-specific activator

**Fig 2:**
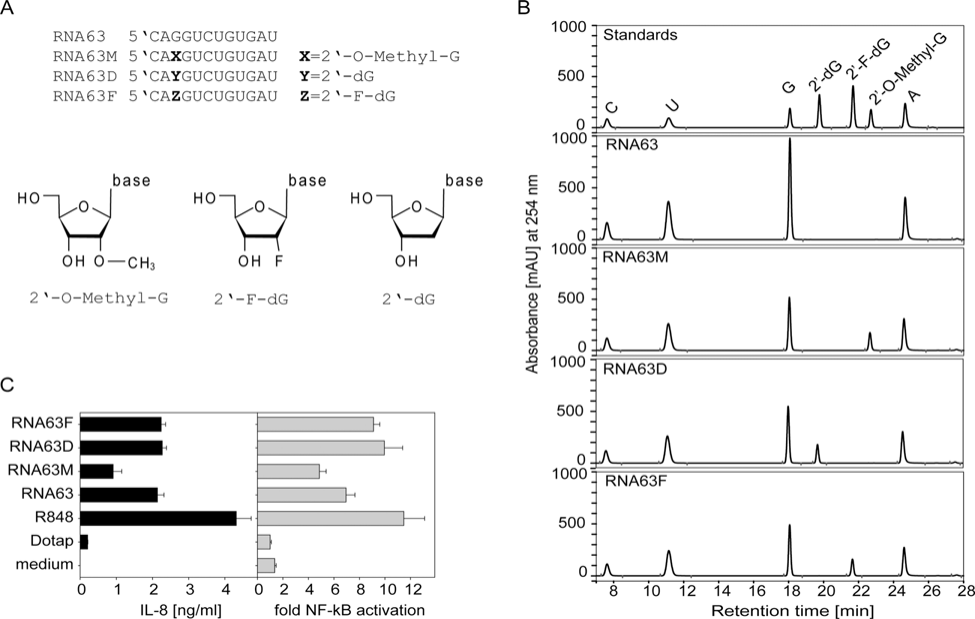
Nucleoside composition of non- and modified synthetic ORN and their TLR8-dependent immunostimulatory potential. (A) Sequence of ORNs used and visualized image of ribose modifications. (B) HPLC analysis of P1 nuclease and phosphatase treated synthetic ORN (RNA63, RNA63M, RNA63D, RNA63F) and corresponding standard nucleosides. (C) HEK-TLR8 cells were stimulated with ORNs as indicated (20 μg/ml RNA63 variants and 5 μg/ml R-848) and 1,5 μM poly-dT. IL-8 secretion or NF-kB activation was measured by ELISA or luciferase assay. One representative experiment of at least three independent experiments is shown (n = 2 ±SD).

To address the TLR-specific activation of 2’-O-methylated RNA we performed genetic complementation experiments with ectopic human TLR-expression in HEK293 cells and extended the analysis to additional 2’-O-modifications at position 3 of RNA63 such as 2’-deoxy (RNA63D) and 2’-fluoro (RNA63F). Similar to 2’-O-methyl, these modifications have been introduced into synthetic RNAs to enhance stability against RNases and therefore enhance knockdown efficiency of siRNA [22,23,32–34]. We confirmed the presence of the modifications within the synthetic RNAs by analysing their nucleoside composition and comparing it to standard nucleosides. Accordingly, HPLC analysis of P1 nuclease / phosphatase treated ORN samples showed the presence of the corresponding modified guanosines (Fig. 2A).

**Fig 3:**
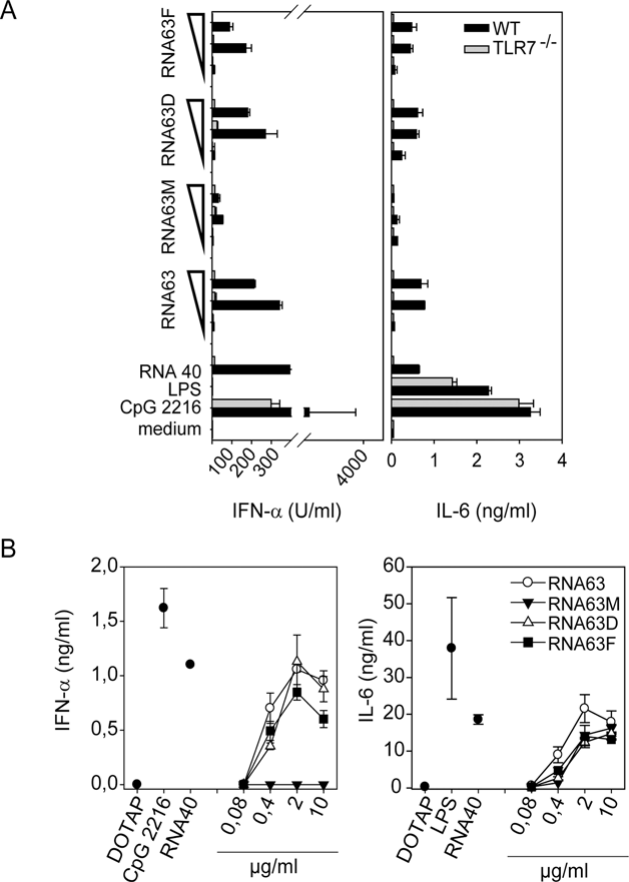
ORN-mediated cytokine production in murine FLT3L DCs and human PBMCs. Murine WT or TLR7-deficient FLT3L DC (A) or human PBMCs (B) were stimulated with RNA63, RNA63M, RNA63D and RNA63F at 10, 2 and 0,4 μg/ml and IFN-α and IL-6 was measured after 20h by ELISA. One representative experiment of three (A) or six (B) independent experiments is shown (n = 2 ±SD).

Loss of hTLR7-induced NF-κB activation by RNA63M could not be studied since TLR7 reconstituted HEK293 cells do not respond to transfected RNA although activation by imidazoquinolines is very effective [10] (and data not shown). For hTLR8 stimulation, poly-dT was added which enhances TLR8-mediated stimulation by RNA in transient transfection systems [16,35]. Of note, hTLR8 expressing HEK293 cells activated NF-κB and secreted proinflammatory IL-8 upon stimulation by RNA63 and RNA63M (Fig. 2B). Since RNA63M induced NF-κB activation and IL-8 production, we conclude that the single 2’-O-methylation does not interfere with TLR8 activation. Since TLR7 activation is abolished by RNA63M (Fig. 1B, C) 2’-O-methylation renders the TLR7/8 ligand RNA63 to a TLR8-specific ligand. Interestingly, 2’-deoxy or 2’-F modifications also had no strong influence on TLR8-mediated NF-κB activation or IL-8 secretion. We further tested the immunostimulatory capacity of these modified ORNs on primary immune cells. In contrast to RNA63M, none of these modifications in RNA63D or RNA63F influenced IFN-α and IL-6 production in murine FLT3-L induced DCs and human PBMCs (Fig. 3A, B) when compared to the cytokine induction induced by RNA63. In summary, the single 2’-O-methylation in a rRNA fragment, but not the 2’-deoxy or 2’-F modifications abolished TLR7-mediated IFN-α production, whereas none of the modifications influenced TLR8-mediated secretion of proinflammatory cytokines.

We therefore conclude that the single natural occurring 2’-O-methylation derived from rRNA at position 1490 converts a TLR7- and TLR8-activating RNA into a specific TLR8 ligand. It is currently unknown how a single 2’-O-methylation within RNA confers this differential effect on TLR7 / TLR8 activation. In the past, binding studies have demonstrated that 2’-O-methylated RNA binds stronger to TLR7 than non-modified RNA [24], but how this interaction translates into a non-stimulatory / antagonistic function of 2’-O-methylated RNA for TLR7 is unknown. Although structural data on TLR8 binding of CL097, a TLR7/8 activating imidazoquinoline, are available [36], structural information on RNA-binding by TLR7 and TLR8 will be necessary to fully understand the effect of 2’-O-methylation on TLR activation.

Although base analoga such as imidazoquinolines and thiazoloquinolone effectively activate TLR7 and TLR8, their side effects potentially pose a problem for systemic application [17–19,37]. Here RNA may prove as safer immune stimulant, since it potentially has less side effects [20]. We provide new information on specific TLR8 activation with 2’-O-methylated RNA and extend the class of TLR8-activating adenosine / uridine rich ORNs [10,15]. However, one disadvantage of RNA mediated immune stimulation may be the need for a cationic transfection reagent such as DOTAP allowing for RNA protection and transport into endosomal vesicles. Interestingly, antimicrobial peptides such as LL37 [38] or other positively charged peptides (e.g. protamine) [39] have been used as carriers for RNA to initiate effective immune stimulation. Their use with RNA63M could specifically stimulate TLR8 and drive proinflammatory cytokine responses.

## Material and Methods

### Ethics Statement

The local ethics committees of Justus-Liebig-University Giessen and Philipps-University Marburg approved the use of human blood samples for this study. For experiments with murine immune cells, mice were sacrificed and tissue / organs removed. These experiments were performed in accordance with the National German welfare law §4 (3) TierSchG and §2 and Annex 2 (TierSchVerV) of the National Order for the use of animals in research and do not need the approval by a local ethics committee. According to the regulations, the number of mice used was reported to the animal welfare officer of the Philipps-University Marburg.

### Reagents

R-848 and type A CpG-ODN2216 (5’-GsGsGGGACGATCGTCsGsGsGsGsGsG) were synthesized by TIB Molbiol, Berlin. Phosphorothioate protected positions of DNA ODNs are indicated by ‘s’. Cell culture grade RNA oligonucleotides RNA63 (CAGGUCUGUGAU), RNA63M (CAXGUCUGUGAU, 'X' depicts 2'-O-Methyl-guanosine), RNA63D (CAYGUCUGUGAU, 'Y' depicts 2'-deoxy-guanosine), RNA63F (CAZGUCUGUGAU, 'Z' depicts 2'-fluoro-deoxy-guanosine) and fluorescence labelled RNA63-Alexa547 and RNA63M-Alexa633 were synthesized by IBA, Göttingen. Anti murine CD40-FITC and CD69-FITC antibody were from BD PharMingen, San Diego, CA. The transfection reagent DOTAP was purchased from Roche, Mannheim, Germany.

### Cells

Human PBMC were isolated from buffy coats by Ficoll gradient centrifugation and seeded at 3x10^5^ cells/well for cytokine induction. FLT-3 ligand induced mixed cultures of murine myeloid and plasmacytoid dendritic cells were grown as described [40]. Murine DCs were seeded at 2x10^5^ cells/well. Human and murine primary cells were cultivated in RPMI (PAN, Aidenbach, Germany) supplemented with 10% fetal calf serum (FCS), 4 mM L-glutamine and 10-^5^ M mercaptoethanol.

### Cell stimulation and staining

For extracellular staining and cytokine induction, human or murine cells were incubated for 14–20 hours with 10 μg/ml RNA63, RNA63M (or titrated as indicated in the figures), 1 μM CpG-ODN2216, 0,1 μg/ml LPS, 0,6 μg/ml RNA40 or 1 μM R-848. Complexation of RNA with DOTAP (Roche, Mannheim, Germany) was performed according to the manufacturers’ recommendation. Briefly, RNA in 50 μl Opti-MEM was combined with 50 μl solution containing 3 μl DOTAP and 47 μl Opti-MEM and incubated for 5 min. 100 μl complete RPMI medium were added and the solution was mixed. 100 μl were used for stimulation of a 96-well that contained primary cells in 100 μl complete medium.

### Cytokine measurement

Concentrations of murine and human IL-6 and IL-8 in the culture supernatants were determined by ELISA according to the manufacturer’s instructions (R&D Biosystems, Wiesbaden for murine IL-6 and human IL-8 and Pharmingen, Leiden, Netherlands for human IL-6). Murine IFN-α was analysed using a PBL Interferon Source (Piscataway, USA) ELISA kit. Concentrations of human IFN-α were determined using mouse monoclonal human IFN-α (PBL Interferon Source) as capture antibody and anti-human IFN-α HRP-Conjugate (eBioscience, San Diego, USA) as detection antibody. The IFN-α standard used was recombinant human IFN-α from PeproTech, Rocky Hill, USA.

### Genetic complementation assays

HEK293 cells stably expressing TLR8 were seeded at 3x10^4^ cells/well in 96-well plates. For luciferase assay, cells were transfected with 2,5 ng NF-κB luciferase reporter-plasmid per well and empty expression vector ad 100 ng. Cells were not subjected to transfection when IL-8 was detected. Stimulation was performed 12h post transfection with 20 μg/ml RNA63, RNA63M, RNA63D, RNA63F and 1 μM R-848 complexed to DOTAP. To enhance TLR8-mediated stimulation, poly-dT-PTO (Metabion, Martinsried, Germany) was added to a final concentration of 1,5 μM. 16–18h post stimulation supernatants were harvested or cells were lysed in 2x Lysis-Juice 2 (PJK GmbH, Kleinblittersdorf, Germany). Luciferase activity was assayed using a Berthold luminometer (Pforzheim, Germany).

### HPLC analysis

Aliquots of RNA equivalent to 10 μg were dissolved in 40 mM ammonium acetate, pH 5.0, containing 3 mM zinc chloride. After addition of 0.25 U nuclease P1 (from Penicillium citrinum, Sigma, Taufkirchen, Germany) per μg RNA samples were incubated over night at 37°C. Thereafter, samples were made alkaline by the addition of TRIS-HCl, pH 8.3 (final concentration 15 mM), containing 1,5 mM magnesium acetate and treated with shrimp alkaline phosphatase (Sigma) and snake venom phosphatase (phosphodiesterase I, Type VI from Crotalus adamanteus, Sigma) at 0.25 U/μg RNA and 0.1 mU/μg RNA, respectively. After incubation at 37°C for 2 hours samples were centrifuged at 30.000 x g for 10 minutes and the supernatant was harvested. HPLC analysis of digests was performed on a Dionex Ultimate 3000 System using a Supelcosil LC18 reverse phase column (250 x 4.6 mm, 5 μm) protected with a C18 guard column (4 x 2 mm) both obtained from Sigma (Taufkirchen, Germany). The mobile phase was: eluent A, 5 mM ammonium acetate, pH 6.0; eluent B, 40% acetonitrile. After injection of the RNA digest separation started with 100% eluent A followed by a 66 minute gradient to 60% eluent B using a flow rate of 0.85 ml/min. Finally the column was subjected to isocratic elution with 100% eluent B for 20 minutes. Detection of the eluted nucleosides was continuously recorded by UV spectrometry at 254 nm.
